# Infrared Laser Heating Reveals a Reversible Phase Transition of Phosphofructokinase 1 in Mammalian Cells

**DOI:** 10.1002/chem.71500

**Published:** 2026-08-03

**Authors:** Morten Mosbach, Sneha Pavanan Kuruvanthara, Lukas Johannknecht, Simon Ebbinghaus

**Affiliations:** ^1^ Lehrstuhl für Biophysikalische Chemie and Research Center Chemical Sciences and Sustainability Research Alliance Ruhr Bochum Germany

**Keywords:** biophysics, condensates, metabolons, phase transitions

## Abstract

Metabolic enzymes can operate in concerted networks, so‐called metabolons. The glucosome is a multi‐enzyme complex that includes enzymes of glycolysis and gluconeogenesis and regulates glucose metabolism in mammalian cells. The mechanism of how glucosomes assemble remains largely unknown. Fluorescence recovery after photobleaching (FRAP) experiments suggest that glucosomes are liquid‐like, but direct evidence of a phase transition mechanism is missing. Here, we developed a novel method to measure the phase transition of condensates in living cells by combining infrared laser heating with confocal microscopy of fluorescently labeled phosphofructokinase 1 liver‐type (PFKL). We showed that glucosomes form and dissolve reversibly in HeLa cells upon temperature modulation, allowing us to determine the condensation temperature. We studied cells under osmotic stress to show that glucosomes retain their phase behavior despite a significant decrease in the mobility of PFKL. We propose that this method is a versatile tool to study the phase behavior of biomolecular condensates, yielding complementary information to well‐established techniques such as FRAP.

## Graphical abstract

In this study, we present a novel method to investigate the phase behavior of biomolecular condensates in living cells. The method complements FRAP experiments, expanding the existing toolbox for characterization of condensates within living cells.

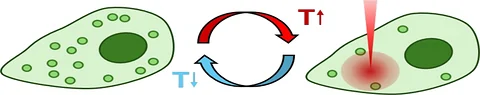

## 1 Introduction

The spatial organization of various cellular processes in metabolism, cell cycle, and cell division relies on biomolecular condensates (BMCs), membraneless organelles based on multivalent protein interactions [1, 2]. BMCs can, for example, increase the local enzyme concentration, while the lack of a physical barrier, such as a lipid membrane, enables molecular exchange with the surroundings [3]. When primarily composed of metabolic enzymes, as seen for the tricarboxylic acid (TCA) cycle [4], the de novo purine biosynthesis [5], or glycolysis [6], BMCs can be specified as so‐called metabolons. The term was initially coined in 1985 by Srere, referring to “a supramolecular complex of sequential metabolic enzymes and cellular structural elements” [7]. The enrichment of multiple enzymes of the same pathway facilitates substrate channeling, where the product of one enzymatic reaction is handed over to the next enzyme. This enables the fast turnover of toxic or labile intermediates and prevents futile cycles driven by metabolic cross‐talk. Thereby, the micro‐compartmentalization enables an increased efficiency of enzymatic reactions but also constitutes a signaling hub for metabolic regulation [8, 9]. Metabolons show key properties of liquid droplets [3], including a spherical shape, the ability to fuse, and molecular mobility, suggesting formation via phase separation [10].

In 2017, Kohnhorst et al. identified clusters of phosphofructokinase 1 liver‐type (PFKL) in mammalian cells, which were termed glucosomes. This enzyme catalyzes the conversion of fructose 6‐phosphate (F6P) to fructose  1,6‐bisphosphate (F1,6BP) and thereby performs the rate‐limiting step of glycolysis [11]. The study further showed colocalization with other rate‐limiting enzymes from glycolysis and gluconeogenesis. Glucosomes are dynamic, membraneless multienzyme assemblies in human cells that help organize glucose metabolism. They can shift glucose flux between energy metabolism and building block biosynthesis in a cluster size‐dependent manner. While medium‐sized clusters of glucosomes direct glucose flux into the pentose phosphate pathway, large‐sized clusters shunt glucose flux towards serine biosynthesis [12]. Their size and assembly state influence whether cells use glucose mainly for energy supply or for building new biomass. This is of special interest, as glucose metabolism is dysregulated in highly proliferative cells, such as cancer cells, where glucose is mainly used for building block biosynthesis even in the presence of oxygen. The size‐function dependency has further implications within the cell cycle. The amount of small‐ and medium‐sized glucosomes significantly increases in G1 phase to meet the metabolic demands, whereas the percentage of cells with large‐sized glucosomes stays consistent throughout the cell cycle [13]. This additional temporal organization of glucosome assemblies indicates a complex underlying regulatory network. One example is the EGF–ERK1/2 signaling pathway, which upregulates the amount of large‐sized glucosomes in cancer cells, therefore functionally promoting glycolysis‐derived serine biosynthesis [14]. Additional studies showed that glucosomes are influenced by a variety of surrounding factors, such as salt concentrations, with hyperosmotic stress altering the composition ratio inside glucosomes [15]. Kang et al. developed a stochastic model in silico studying the contribution of the biological properties of PFKL to condensate formation, focusing on the intermolecular interactions [16]. Nonetheless, the mechanisms of how glucosomes assemble remain largely unknown [17].

To investigate if glucosomes form by phase separation, we measured their temperature‐dependent phase behavior inside mammalian cells. A phase separation mechanism, governed by thermodynamic principles, would lead to a phase diagram with a reversible, temperature‐dependent phase transition. Upon an increase in temperature, both mixing and demixing of two phases were observed in vitro as well as in cellular systems [18, 19]. Heat shock factor 1 shows a lower critical solution temperature (LCST)‐type phase transition, with phase separation occurring at elevated temperatures [20]. The heating of P granules, on the other hand, resulted in their dissolution, displaying an upper critical solution temperature (UCST) [21]. Here, we introduce a novel technique to heat living mammalian cells with subcellular resolution, probing the local temperature response of individual condensates as visualized by labeled PFKL. We show that glucosomes form reversibly by phase separation and determine their condensation temperature under different cellular conditions.

## 2 Results and Discussion

### 2.1 PFKL‐mEGFP Foci Show Properties of Biomolecular Condensates

We detected the formation of glucosomes by labeling the PFKL enzyme [11] using an mEGFP‐tag [22]. The tagged protein was transiently transfected into HeLa cells. Imaging at 18°C using confocal microscopy, cells displayed foci within the cytosol with an average size of 0.89 µm^2^. As a control, we expressed the mEGFP‐protein without the PFKL fusion, which resulted in homogeneous fluorescence in the cytosol (Figure S1). Further, the platelet isoform of the PFK enzyme (PFKP), was investigated, which does not form cytosolic condensates [23]. Indeed, we did not observe the formation of foci for this isoform (Figure S2). Next, we tested if the observed foci show properties previously reported for glucosomes. We could promote glucosome formation via glycolytic inhibition with 2‐Deoxy‐D‐glucose (2DOG) as previously described for glucosomes (Figure S3) [12]. We then verified that foci are not stress granules that sequester the overexpressed PFKL‐mEGFP protein. We used a HeLa cell line stably expressing the fused in sarcoma (FUS) protein labeled by mCherry [24] as a marker for stress granules [25, 26]. We did not find colocalization of FUS‐mCherry and PFKL‐mEGFP (Figure S4). We then tested if the foci are mobile, a hallmark of biomolecular condensates [27, 28]. We used fluorescence recovery after photobleaching (FRAP) measurements to determine the mobile fraction (Figure 1a). FRAP experiments of condensates at 18°C showed a mobile fraction of 0.56 ± 0.16 and of 0.71 ± 0.14 at 37°C. These values are comparable to other BMCs [29, 30, 31] and confirm the liquid‐like character of the foci. Further evidence of the liquid‐like character of the foci comes from the observation of fusion events of condensates in the cell (Figure 1b) [3, 32]. In summary, we could show that PFKL‐mEGFP protein is a suitable marker to observe glucosomes, and the results provide further evidence that glucosomes show properties of liquid‐like BMCs.

**FIGURE 1:**
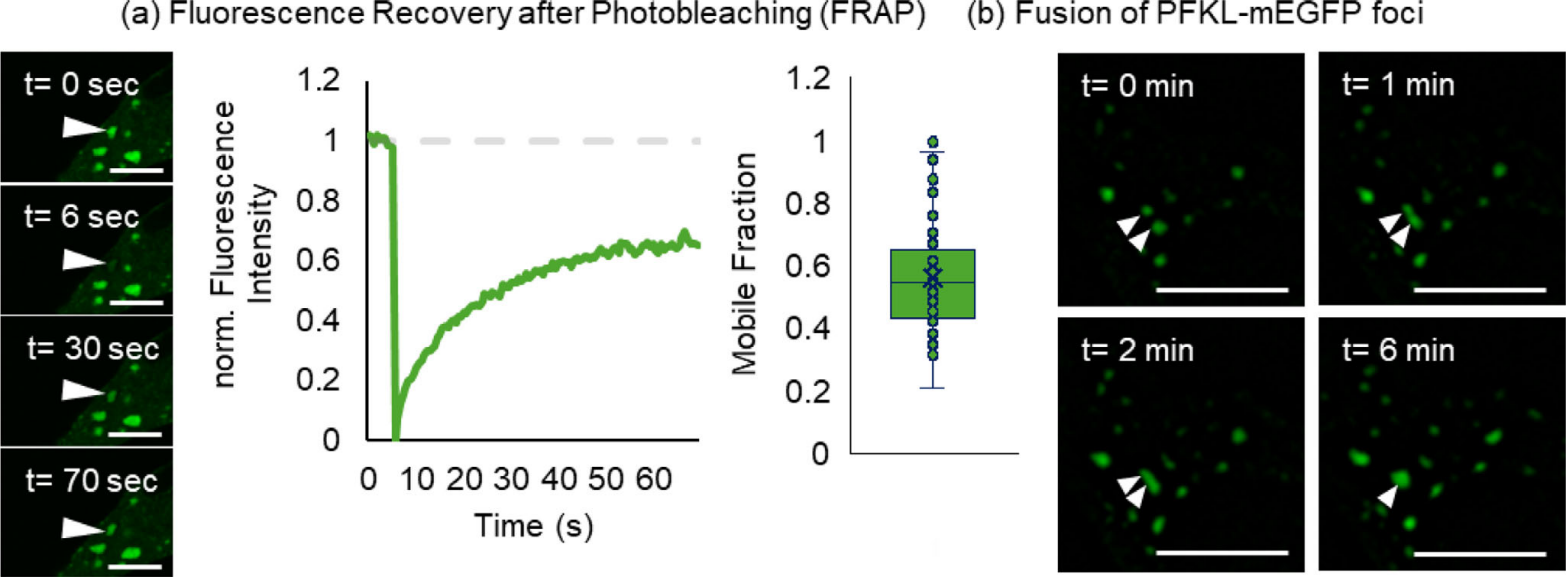
Liquid‐like properties of glucosomes. (a) Fluorescence recovery after photobleaching (FRAP). *Left*: Representative images of a selected condensate (white arrow) before bleaching at *t = 0 sec*, followed by bleaching by an intense 488 nm laser pulse at time *t = 6 sec*. The recovery of the fluorescence was monitored over 70 seconds (*middle*), and the mobile fractions were calculated for N_condensates_ =  77 (*right*). The boxplot box represents the interquartile range (IQR), with whiskers extending to the minimum and maximum data points within 1.5 times the IQR. The mean is indicated by an ‘x’ marker, and the median is shown by a horizontal line within the box. (b) Fusion event of two foci spontaneously observed in a cell. Two separate foci (*t = 0 min*) merge into one within 6 minutes. Scale bars: 10 µm.

### 2.2 Combining Confocal Imaging and IR Laser Heating to Investigate the Phase Transition

We aimed to measure the fast temperature‐induced phase transition of glucosomes independent of processes that occur on slower timescales, for example, the overexpression of heat shock proteins [33]. If glucosomes are formed by phase transition following thermodynamic principles, condensates are expected to dissolve reversibly with increasing temperature (if showing an UCST behavior [34]) with a well‐defined condensation temperature *T_cond_*. (Figure 2a).

**FIGURE 2:**
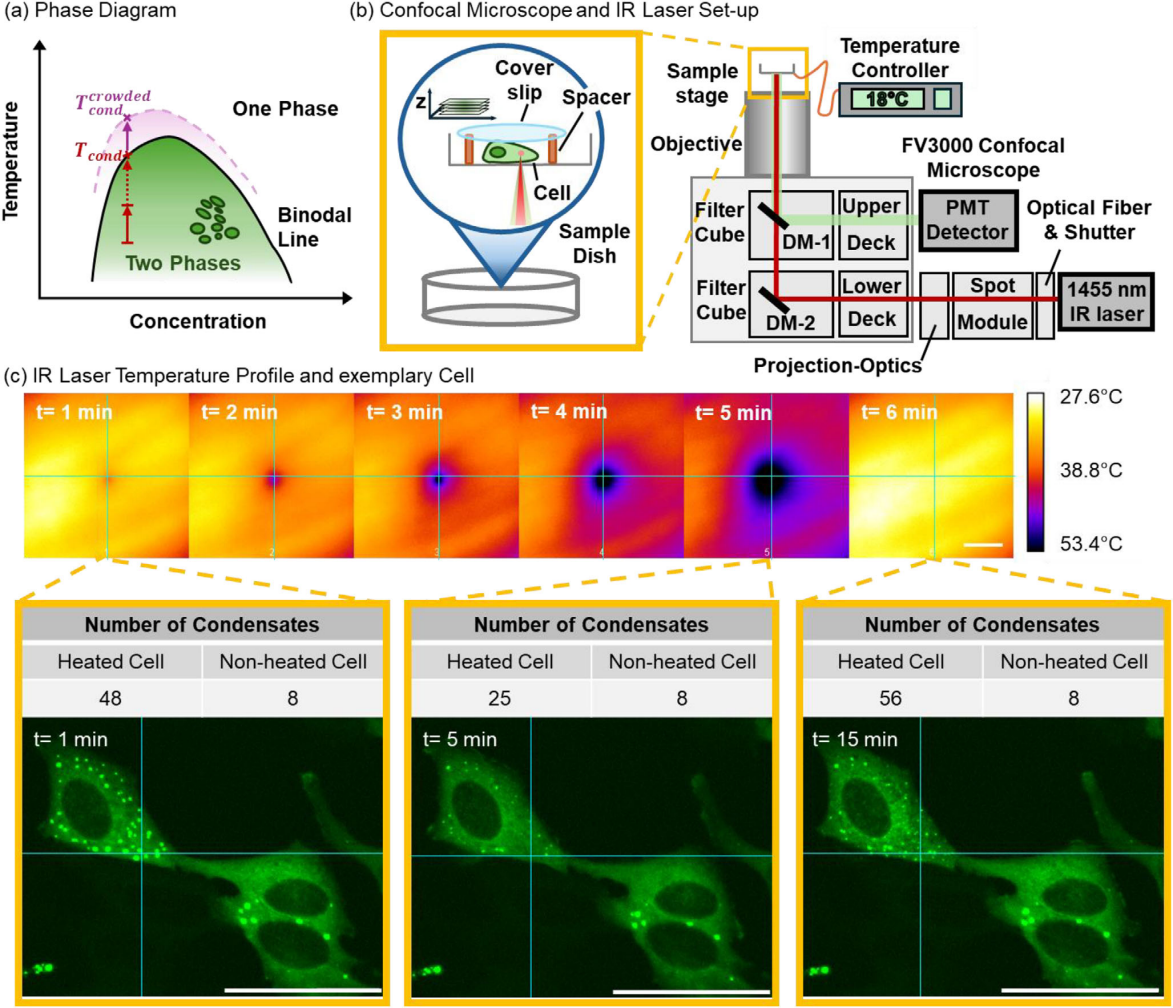
Temperature‐induced phase transition of glucosomes. (a) Illustration of a binary phase diagram showing UCST behavior. Increasing the temperature at an invariant concentration leads to condensate dissolution at *T_cond_*. Different cellular conditions shift the binodal line and change *T_cond_*. (b) Implementation of IR laser heating on a confocal platform. The IR laser is focused onto the cells using a separate deck of the confocal microscope. (c) *Top*: Temperature profile and its evolution in time induced by IR laser output power modulation [36]. *Bottom*: Representative images of HeLa cells heated according to the temperature profile shown on *top*. The temperature profile and the cell images can be overlaid by the crossing of the blue lines and scale bars to determine the temperature at each position of the cell. Scale bars: 50 µm.

To build the technique, we integrated an infrared (IR) laser (1455 nm) into a confocal laser scanning microscope using a distinct optical deck. The laser beam was projected from the optical fiber onto the sample via the projection optics after passing a switchable optical module for focusing (Figure 2b). The IR laser and the visible light were split via a dichroic mirror, reflecting the visible light towards the detectors while letting the IR laser pass through. The IR laser output power was modulated in time to control heating rates and focused onto the cells to heat only a single cell at a time (Figure 2c). This allowed us to probe condensates with subcellular resolution and investigate single cells on a cell culture dish. The temperature gradient was induced by a gradual increase in the laser power until *t = 5 min* and the following shut‐off for rapid cooling (Figure S5). This brief heating interval was chosen to minimize interference from cellular adaptation mechanisms, such as the heat shock‐induced upregulation of molecular chaperones, which typically peaks after a delay of around 40 min [35]. The temperature was estimated using Rhodamine B fluorescence as described in earlier protocols [36] (see Materials and Methods for details). The heating is centrosymmetric and is governed by the Gaussian heating profile of the IR laser (heating water at 1450 nm; absorption coefficient of water approximately 2 mm^−1^[37]), as well as the heat dissipation by the water and the sample dish substrate [38].

We then exposed cells with formed glucosomes to the temperature profile shown in Figure 2. We observed that glucosomes dissolved upon heating, as their number, as well as their size, decreased (Figures 2c and 3). The process was reversible (Figure 3a,b), even for a second cycle of temperature‐dependent formation and dissolution within the same cells (Figure S6), which is a characteristic of a thermodynamic phase transition [39, 40]. Furthermore, we investigated the average area of the single detected condensates, which decreased upon heating from 0.89 µm^2^ to around 0.60 µm^2^ but recovered only to 0.67 µm^2^ (Figure 3c). The dissolution of larger condensates upon heating resulted in a larger number but a smaller average area of reformed glucosomes during the following cooling period. FRAP experiments before and after heating showed that the condensates maintain liquid‐like material properties (Figure S7). We further analyzed the kinetics of droplet formation and dissolution. To assess the relaxation process leading to the new thermodynamic equilibrium at elevated temperature, we performed rapid temperature‐jump experiments (1.8°C ± 0.13°C heating jump). These experiments allowed us to determine the kinetics of condensate dissolution. For 1.8°C temperature jumps applied at 23°C, we calculated an average relaxation time constant τ= 8.7 ± 2.3 s for condensate dissolution (Figure S8), corresponding to 95% equilibration after 26.1 s. Given the maximum heating rate of β= 25.8°C per 5 min in the experiments using continuous heating, that is, 0.086°C/s, in the center of the laser focus and a total heating time of 5 min, we conclude that the system is largely equilibrated at the respective temperatures. We further tested whether *T_cond_* depends on the heating rate, which should not be the case if the system is at equilibrium. Because the heating amplitude varies across different positions of the cell, the local heating rate also changes. Indeed, we found no correlation between *T_cond_* and heating rate (Figure S9).

**FIGURE 3:**
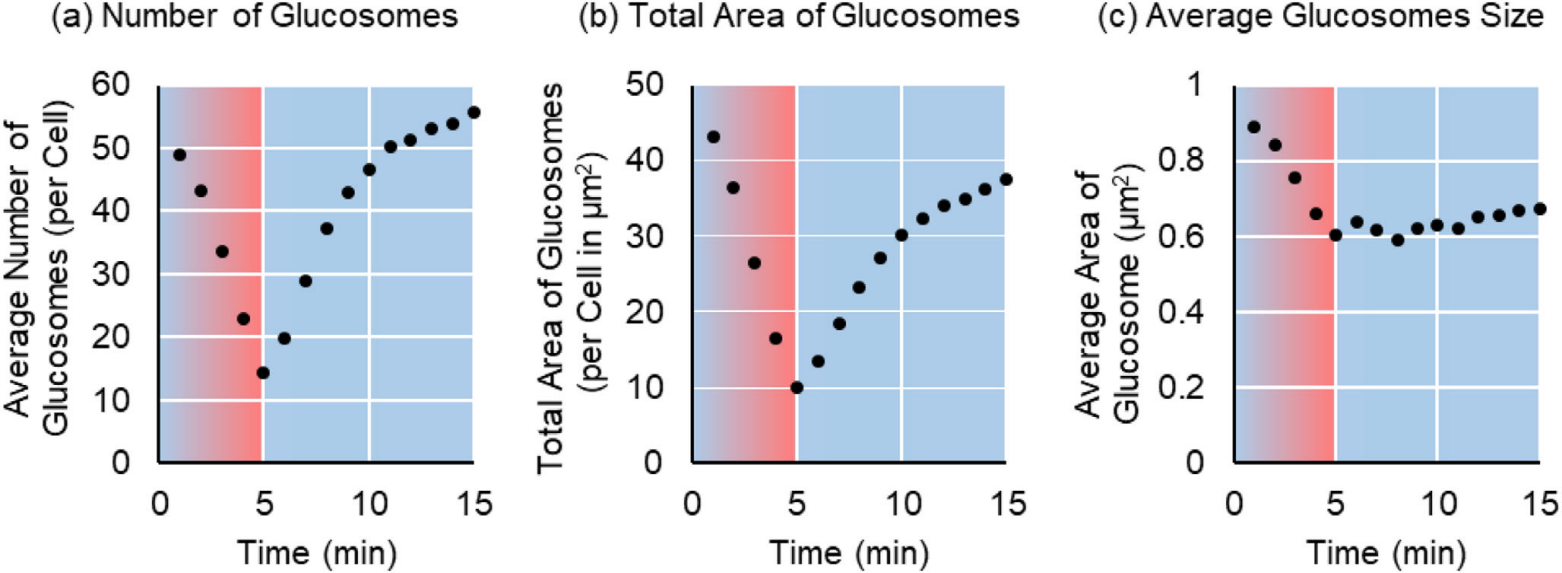
Glucosome dissolution and formation upon temperature changes using the temperature profile shown in Figure 2 (temperature increase and decrease illustrated by red and blue background colors). (a) Average number of glucosomes per cell. (b)Total area of glucosomes. (c) Average size of glucosomes. Numbers show the average of 1324 analyzed glucosomes from 27 cells over more than three repeats.

As temperature increases, the concentrations of molecules in the droplet and dilute phases change according to the phase diagram, resulting in smaller droplets or droplets of reduced size [41], which fully dissolve at *T_cond_*. Since this point cannot be reached in our experiments (as higher temperatures would be detrimental to cells), we fitted the temperature response of the droplets to a linear function and extrapolated the curve to the temperature at which the droplet area equals zero (further discussed in the Supporting Information) [21]. Therefore, each droplet was tracked during heating (see Materials and Methods for details). Because the analyzed *T*_*cond*_​ was only weakly dependent on droplet size (Figure S10), we primarily analyzed larger droplets that could be tracked at three or more temperatures (Figure 4). The analysis yields x‐intercepts with an average estimated *T_cond_* of 57.9 ± 9.2°C (N_cells_ = 5; N_condensates_ = 72) for glucosomes induced at 18°C. The error represents the standard deviation of individual droplet x‐intercepts. This error is determined by the fit errors of the individual droplets, since the comparison of individual cells leads to a smaller standard deviation of 2.5°C. Next, we investigated glucosomes in the presence of osmolytes and electrolytes, both conditions known to influence the material properties of condensates [42]. To monitor the mobility of glucosomes, we conducted FRAP measurements. We added NaCl (6 and 12 g/L) as well as the osmolyte sucrose (50 and 100 mM) to the imaging medium, leading to osmotic stress of the cell [43]. We observed that the mobile fraction decreased from 0.56 ± 0.16 to 0.28 ± 0.13 for NaCl and to 0.08 ± 0.02 for sucrose addition (Figure 5a). Such a decrease in mobility is commonly interpreted as a “solidification” of condensates, possibly indicating a liquid‐solid phase transition [44, 45]. Accordingly, we observed an increase in *T_cond_*, revealing a stabilization of the droplets (Figure 5b). The unchanged droplet formation kinetics (Figure S11) indicate that the shift can be attributed to a thermodynamic effect. Further, the reversibility of the phase transition is maintained despite the apparent loss of mobility of glucosomes (Figure S12).

**FIGURE 4:**
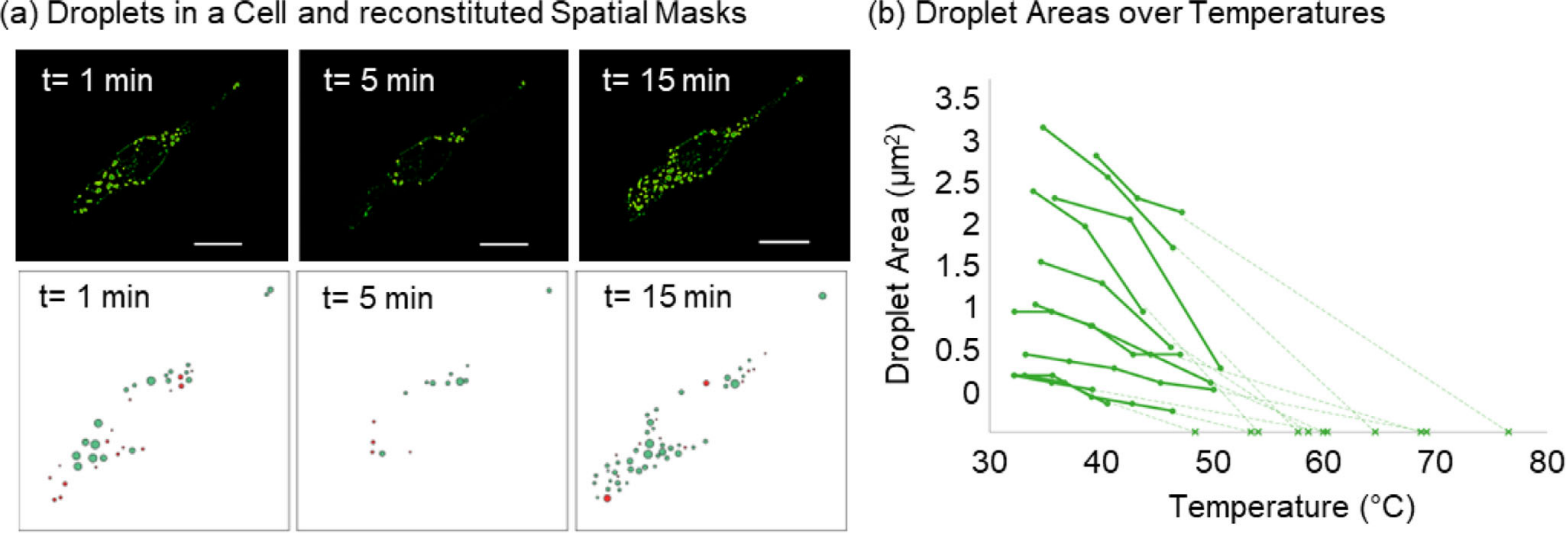
Droplet dissolution and formation in HeLa cells transfected with PFKL‐mEGFP induced by the heating profile shown in Figure 2c. (a) *Top*: Background subtracted microcopy images of a HeLa cell at the beginning (*t = 1 min*), during (*t = 5 min*), and after (*t = 15 min*) heating. *Bottom*: Tracking of PFKL‐mEGFP droplets with time (see Materials and Methods for details). Droplets that dissolve during heating are shown in red. Scale bar: 20 µm. (b) Plot of single droplet areas against temperature. Dotted lines represent linear fits which were extrapolated to determine the interception of the *x*‐axis.

**FIGURE 5:**
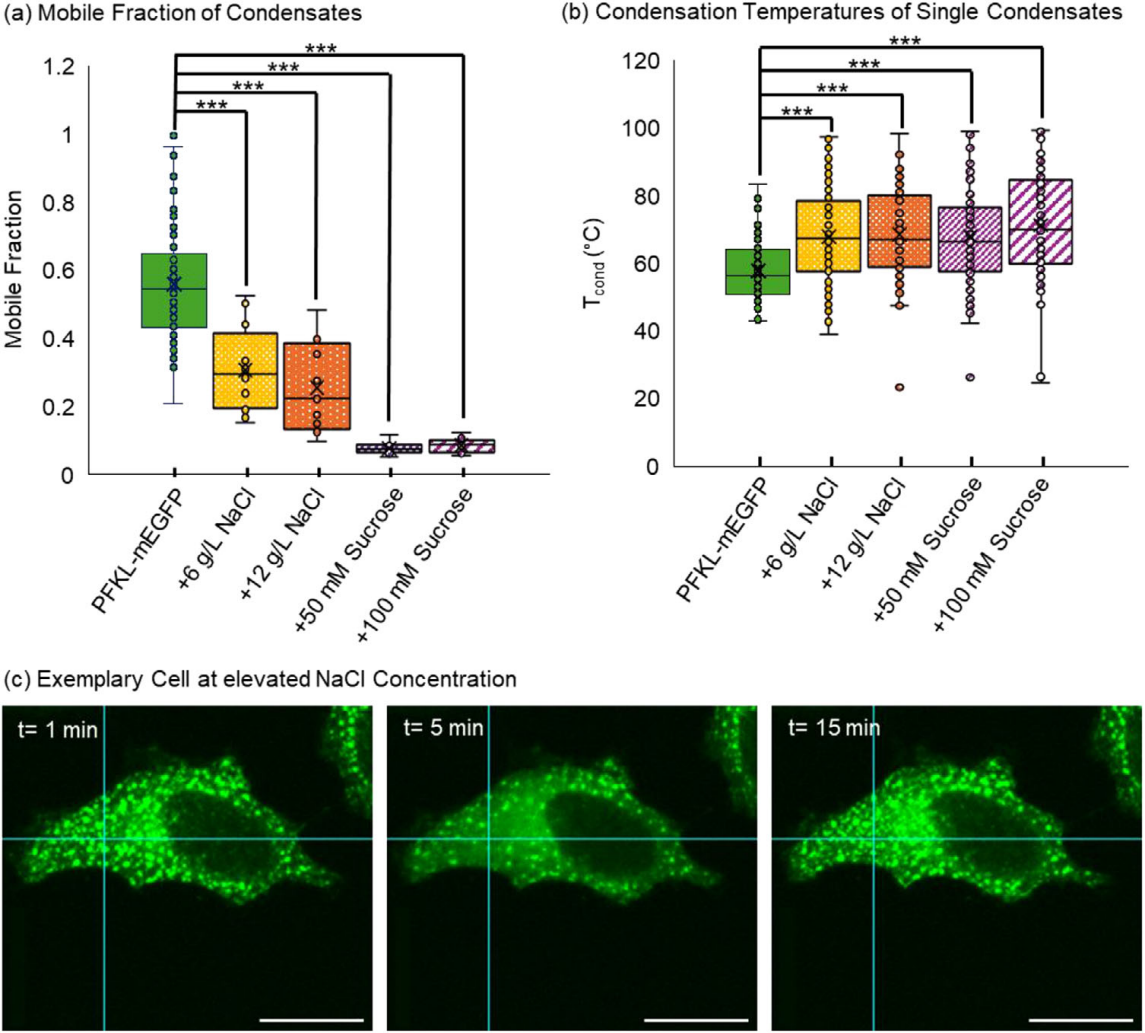
Influence of NaCl and sucrose on (a) the mobile fractions and (b) condensation temperatures of PFKL‐mEGFP condensates. Circles represent single condensate measurements. Boxplots show boxes representing the interquartile range (IQR), with whiskers extending to the minimum and maximum data points within 1.5 times the IQR. The mean is indicated by an ‘x’ marker, and the median is shown by a horizontal line within the box. Significance was tested by an independent two‐sample *t*‐test. For mobile fractions and condensation temperatures, all conditions showed significance with *p*‐values <0.0005 compared to PFKL‐mEGFP. (c) Exemplary image of a HeLa cell transfected with PFKL‐mEGFP with an additional 12 g/L of NaCl. Images represent the same cell at the beginning (*t = 1 min*), during (*t = 5 min*), and after (*t = 15 min*) the heating. Scale bar: 20 µm.

## 3 Conclusion

IR laser heating in combination with confocal imaging is a promising tool to characterize phase transitions in living cells. This method provides high spatial and temporal flexibility for applied temperature changes, which is advantageous compared to stage heating, where temperature changes are only achievable slowly and globally [21]. The IR laser can be readily implemented into a confocal microscope using the same optical path as for confocal imaging. The laser allows for inducing circularly shaped temperature gradients that can be adjusted spatially by focusing (down to the diffraction limit) and defocusing of the laser. Different heating spot sizes can be employed depending on whether the aim is to investigate multiple cells simultaneously or to heat a single area repetitively, even within one sample dish. The heating rate or temperature profile can be specifically tailored by directly modulating the IR laser output power [46]. This is crucial, especially for the investigation of different condensates without exposing them to non‐physiological temperatures for extended periods, potentially eliciting heat shock responses [33].

Our findings indicate that glucosome formation and dissolution are governed by thermodynamic principles. Similar to P‐granules [21], we found that glucosomes form and dissolve reversibly. Glucosomes were less mobile after NaCl addition, and immobile after the addition of sucrose, as shown by FRAP measurements. However, the temperature‐dependent experiments showed that the condensates still maintain liquid‐like character, being able to dissolve and reform reversibly while displaying higher average condensation temperatures. The reason for the apparent discrepancy between the experiments is that molecular interactions responsible for material properties of droplets are not necessarily the same interactions that govern droplet assembly [47]. The addition of salt as well as sucrose causes osmotic stress, leading to a shrinking of cell volume and concentration of its biomolecular content [48]. This increases the strength of intermolecular interactions [43, 49, 50], which might lead to a decrease in biomolecular mobility but not necessarily imply that the liquid‐like character of the condensates is lost.

In summary, temperature‐dependent in‐cell studies of condensates yield valuable and quantitative information about their reversibility and phase transition process. Thereby, the method complements FRAP experiments, expanding the existing toolbox for characterization of condensates within living cells.

## 4 Methods

### 4.1 Cell Culture and Transfection

Human cervix adenocarcinoma HeLa cells (DSMZ, ACC 57, RRID: CVCL_0030) were maintained in Dulbecco's Modified Eagle 306 Medium (DMEM)(Thermo Fisher, 11965092) supplemented with 10% fetal bovine serum (FBS)(Thermo Fisher, A5670701) and 1% penicillin‐streptomycin (Thermo Fisher, 15140122) and kept in an incubator at 37°C, 5% CO_2_, and 95% humidity. For imaging, cells were detached using trypsin‐EDTA (Merck, T3924‐500ML) and seeded into glass‐bottomed 35 mm Petri dishes (WPI, FD35‐100). After reattachment of the cells overnight, transfection was carried out using Lipofectamine 2000 (Thermo Fisher, 11668019) and Opti‐MEM‐I reduced serum medium (Thermo Fisher, 31985062). After incubation for 4.5 h, the cells were placed back into normal growth media. Imaging was performed around 18–24 h post transfection.

### 4.2 Confocal Microscopy Combined With IR Laser‐Induced Heating

For imaging, cell medium was replaced with Leibovitz's L‐15 (Thermo Fisher, 21083027) medium supplemented with 30% FBS. For the experiments with electrolytes or osmolytes, the basic medium was supplemented with 6 or 12 g/L of additional NaCl or 50 and 100 mM sucrose. For incubation with glycolytic inhibitor 2‐Deoxy‐D‐glucose (2DOG)(Carl Roth, CN96.1), the imaging medium was supplemented with 25 mM, and the cells were incubated for at least one hour prior to imaging. Cells were washed with DPBS (Thermo Fisher, 14190144) before 33 µL of the imaging medium (with or without the additives) was added, and the cells were subsequently sandwiched between a coverslip (Carl Roth, P233.1) held by a spacer (Sigma–Aldrich, GBL654004‐100EA). The prepared samples were placed in the OkoLab temperature chamber for at least 20 min to achieve temperature equilibration before imaging. Imaging was performed using a confocal laser scanning microscope (FV3000, Olympus) with a 60X water objective lens (UPLSAPO 60XW, Olympus, Japan) with a numerical aperture of 1.2. Laser intensity was set to 1% of the 488 nm laser for imaging of the mEGFP‐based constructs. For IR‐induced heating, a laser with 1455 nm wavelength was used. The IR diode laser was operated in the continuous wave (cw) mode. The additional IR laser unit was mounted to the confocal microscope by Rapp OptoElectronic GmbH. Z‐stacks over a range of 11 µm were captured encompassing the whole cell. Images were analyzed as average intensity z‐projections, which were background‐subtracted via a Gaussian blur filter, before the integrated „Analyze particles″‐tool of FIJI was used to assess the number, total size, and average size of condensates based on an intensity threshold value that needed individual adjustment for each image. Adequate detection was confirmed by eye. A particle size threshold of 0.2 to 5 µm^2^ was applied for condensate detection.

### 4.3 Fluorescence Recovery After Photobleaching (FRAP)

The FRAP experiments were carried out on a confocal laser scanning microscope (FV3000, Olympus) with a 60X water objective lens (UPLSAPO 60XW, Olympus, Japan) with a numerical aperture of 1.2. Unless stated otherwise, cells were incubated at 18°C inside an OkoLab temperature chamber for at least 20 min prior to imaging. Approximately 2 frames per second were captured. ROIs were defined around the bleached condensate, a second condensate as a reference, and a third region next to the cell as background. For the bleaching pulse, the laser intensity of the 488 nm laser was set to 100%. 10 frames (∼5 sec) were collected before bleaching for baseline generation. Followingly, the recovery was observed for about 65 s. After background subtraction from both the FRAP region and the reference region, the time‐dependent mean intensity of the fluorescence signal was normalized according to the easyFrap protocol [51] and the normalized intensity $I{\left(t\right)}_{norm}^{fullscale}$ was fitted (using Matlab) to a single exponential model to obtain the mobile fraction:

$$\;I{\left(t\right)}_{norm}^{fullscale}=I_{0}\;-a*\;e^{-\beta *t}$$

*I*_0_ refers to the mobile fraction of mEGFP and *β* to the rate of recovery.

### 4.4 Analysis of Condensation Temperature

Z‐stacks were captured every minute during 5 minutes of IR heating. Droplets were tracked by registering the center of mass X and Y values across the different time points as a typical straightforward localization approach used in biophysics [52]. Spatial masks of the droplets were projected onto a template consisting of three average z‐projections of RhB measurements. The mean fluorescence intensity of the RhB solution was measured at the positions of the droplets. The temperature dependency of RhB therefore allowed to estimate the temperature each droplet was exposed to. Following, droplets were tracked using an AI‐assisted self‐scripted MATLAB code based on a nearest‐neighbor approach as commonly used to track objects throughout a time‐series of images [53]. Only droplets tracked over three out of five consecutive timepoints of the heating period were considered for evaluation. A linear function was fitted to the droplet sizes to determine the x‐intercept that corresponds to a droplet size of 0. Only extrapolations with an R‐square value above 0.8 were plotted and considered for condensation temperature calculation. Furthermore, intercepts below 23°C, as well as above 100°C, were excluded. Droplet tracking was validated by comparing original images to tracking masks generated by the analysis script based on input areas and X and Y coordinates.

### 4.5 Rhodamine Temperature Calibration

The temperatures induced by the IR laser were measured using the temperature‐dependent fluorescence [54] of RhB (100 µM) following previously established protocols [55]. From the average intensity of z‐projections, the temperature could be acquired by the following relationship: [56]

$$T\;\left(F\right)=149.15-317.84*F+323.41*F^{2}-131.84*F^{3}$$

where T is the temperature, and F is the RhB fluorescence intensity normalized by the average intensity value at 23°C.

### 4.6 Statistical Analysis

Unless stated otherwise, plots represent mean values for the given number of replicates. Boxplots show boxes representing the interquartile range (IQR), with whiskers extending to the minimum and maximum data points within 1.5 times the IQR. The mean is indicated by an ‘x’ marker, and the median is shown by a horizontal line within the box. Significance was tested by independent two‐sample *t*‐tests with asterisks indicating *p*‐values: ^*^:*p* <0.05, ^**^:*p* <0.005 and ^***^:*p* <0.0005.

## Conflicts of Interest

The authors declare no conflicts of Interest.

## Supporting information




**Supporting File 1**: chem71500‐sup‐0001‐SuppMat.docx

## Data Availability

The data that support the findings of this study are available from the corresponding author upon reasonable request.
