# Supplementary material for: Infrared Laser Heating Reveals a Reversible Phase Transition of Phosphofructokinase 1 in Mammalian Cells
Source: Chemistry. 2026 Aug 3;32(40):e71500. doi: 10.1002/chem.71500 (PMC13716000; doi:10.1002/chem.71500)
Supplement: Supplementary file 1 [file CHEM-32-e71500-s001.docx]

**Supporting Information**

**Infrared Laser Heating Reveals a Reversible Phase Transition of Phosphofructokinase 1 in Mammalian Cells**

Morten Mosbach^[a]^, Sneha Pavanan Kuruvanthara^[a]^, Lukas Johannknecht^[a]^ and Simon Ebbinghaus*^[a]^

[a]	Lehrstuhl für Biophysikalische Chemie and Research Center Chemical Sciences and Sustainability

Research Alliance Ruhr

Ruhr University Bochum

Universitätsstraße 150, 44801 Bochum, Germany

E-mail: [simon.ebbinghaus@rub.de](mailto:simon.ebbinghaus@rub.de)

**The supporting information contains Supplementary Figures S1-12 (pages 2-13)**

Text 1: Discussion of the linear fitting of the temperature dependence of condensate sizes to determine *T_cond_* (page 14)

**Figure S1.** Representative images of HeLa cells expressing PFKL-mEGFP showing condensate formation (*upper row*) and the same genetic construct without the PFKL fusion enzyme leading to a homogeneous fluorescence of mEGFP throughout the cytosol (*lower row*). Imaging was done using a confocal laser scanning microscope (FV3000, Olympus) with a 20X objective lens and 5% laser intensity of the 488 nm laser for imaging of the mEGFP-based constructs. Scale bars: 50 µm.

**Figure S2.** Representative image of a HeLa cell expressing PFKP-mEGFP showing a homogeneous distribution of fluorescence. Scale bar: 20 µm.

**Figure S3.** Representative images of HeLa cells transfected with PFKL-mEGFP without (*left*) and with (*right*) 2-Deoxy-D-glucose at 37°C. Imaging was done using the Leica THUNDER Imager 3D Cell Culture & Infinity Laser Scanner with a 40X objective lens and a numerical aperture of 0.6. For excitation, the LED light source 475 nm LED was used with an exposure time of 200 ms. Scale bars: 50 µm.

**Figure S4.** Representative confocal image and zoom-in (*right*) of HeLa cells stably expressing FUS-mCherry as a marker for stress granules (red) transiently transfected with PFKL-mEGFP construct (green). Scale bar: 20 µm.

**Figure S5.** Plot of IR laser intensity over time for the generation of temperature profile as shown in Figure 2c.

**Figure S6.** Glucosome dissolution and formation upon temperature changes using the temperature profile shown in Figure 2 (temperature increase and decrease illustrated by red and blue background colors) using two cycles of heating and cooling. Shown is an average of 3 cells.

**Figure S7**. Comparison of the mobile fraction of mEGFP-PFKL transfected cells (a) before and (b) after heating in normal medium and upon addition of NaCl or sucrose. Circles represent single measurements. Boxplots show boxes representing the interquartile range (IQR), with whiskers extending to the minimum and maximum data points within 1.5 times the IQR. The mean is indicated by an ‘x’ marker, and the median is shown by a horizontal line within the box. Significance was tested by independent two-sample *t*-test. All conditions showed significance with *p*-values <0.0005 compared to PFKL-mEGFP. No significant changes in mobile fractions were detected upon heating for any of the conditions.

**Figure S8.** Estimation of equilibration rate of glucosome area upon temperature jump of 1.8 ± 0.13°C starting from 23°C. Depicted is an average of the total glucosome area per cell for N_cells_= 5 normalized to the pre-temperature jump value. For the temperature jump, cells were exposed to IR laser (λ = 2200 nm, m2k laser, GmbH, Germany) and imaged using a Zeiss AxioObserver Z1 inverted widefield fluorescence microscope with 40X objective (NA = 0.95, Zeiss) at one frame per second (1 fps) over a duration of 50 sec. Dashed line indicates single exponential fit yielding the time constant $\tau$*=* 8.7 ± 2.3 s.

**Figure S9**. Distribution of *T_cond_* throughout a single cell. (a) Representative confocal image of HeLa cell displaying PFKL-GFP condensates with the IR laser beam being centered at the blue line intercept. (b) Spatial map of *T_cond_* determined at different positions within the cytosol represented by a false-color temperature scale (*right*). Scale bar: 20 µm.

**Figure S10.** Area of condensates plotted against *T_cond_*. Only a weak dependence was evident (R = 0.132, N_condensates_ = 495). Black dashed line shows linear fit of all condensates analyzed (red points).

**Figure S11.** Analysis of glucosome area recovery after heating as shown in Figure S12. The IR laser was shut off at *t= 5 min*. Shown is an average of the total glucosome area per cell during 10 minutes of cooling normalized to pre-heating value (*t= 1 min*). Shown is the average of 587 analyzed condensates from 4 cells for the addition of 6 g/L and 1113 analyzed condensates from 7 cells for the addition of 12 g/L NaCl. Average of 1324 analyzed glucosomes from 27 cells for control without NaCl addition. Dashed lines indicate the corresponding single exponential fits applied to fit half-life values and corresponding fit errors of 3.0 ± 0.01 min for control, 3.1 ± 0.01min for +6 g/L NaCl and 2.7 ± 0.01 min for +12 g/L NaCl.

**Figure S12.** Different parameters of glucosomes including (a) the average number per cell and (b) the total area of glucosomes within one cell during the heating (red shading) and following cooling period (blue shading). Average of 587 analyzed condensates from 4 cells for additional 6 g/L and 1113 analyzed condensates from 7 cells for additional 12 g/L NaCl.

SI Text 1: Discussion of the linear fitting of the temperature dependence of condensate sizes to determine *T_cond_*

Figure S10 reveals only a minor dependency between the condensate area and *T_cond_*. This is in agreement with our estimation showing neglectable influences of Gibbs-Thomson effect on glucosomes:

For an estimate, we used the Gibbs-Thomson equation assuming a molecular weight (based on monomer amino acid sequence) of 85 kDa and a molecular volume $v$ (assuming standard protein specific density of ρ = 1.35 g/cm^3^ ^[1]^) of:

$$v= \frac{M}{N_{A}* \rho}\approx4.18*{10}^{-25} m^{3}$$

Further, we used surface tensions γ between 5 – 50 µN/m ^[2]^ and a temperature of T= 310 K. We calculate the capillary length $l_{c}$ as:

$l_{c}= \frac{2\gamma\nu}{k_{B}T}$ ^[3]^

where k_B_ is the Boltzmann constant. This yields:

$c\left( r \right)= c_{\infty}*\left( 1+ \frac{l_{c}}{r} \right)$ ^[4]^

where $c\left( r \right)$ is the coexistence concentration for a droplet in respect to its radius $r$ and $c_{\infty}$ is the coexistence concentration for a flat interface. Table 1 summarizes the calculations for the entire area range of droplets analyzed with different surface tensions reported for biomolecular condensates:

**Table 1.** Effect size of Gibbs-Thomson effect on droplet coexistence concentrations

| **Droplet Area (μm^2^)** | **Radius (μm)** | **Impact for γ = 5 μN/m** | **Impact for γ = 50 μN/m** |
| --- | --- | --- | --- |
| 0.2 | 0.25 | 0.39% | 3.91% |
| 5 | 1.26 | 0.08% | 0.77% |
|  |  |  |  |

Comparing the smallest to largest droplets, the difference in effect size is 0.31% (0.39% - 0.08%) for a surface tension γ of 5 µN/m and 3.14% for γ = 50 µN/m. Within the range of analyzed droplets (Area between 0.2 - 5 μm^2^), the effect of surface curvature appears neglectable. Given the relatively small change in equilibrium concentrations predicted by the Gibbs-Thomson equation, a linear approximation, as applied by Fritsch et al. for determining the condensation temperature, seems appropriate.^[3,5]^

**References**

[1]	H. Fischer, I. Polikarpov, A. F. Craievich, “Average protein density is a molecular-weight-dependent function” *Protein Science* **2004**, *13*, 2825–2828.

[2]	N. Sanfeliu-Cerdán, M. Krieg, “The mechanobiology of biomolecular condensates” *Biophys Rev (Melville)* **2025**, *6*, 011310.

[3]	C. A. Weber, D. Zwicker, F. Jülicher, C. F. Lee, “Physics of active emulsions” *Rep. Prog. Phys.* **2019**, *82*, 064601.

[4]	C. F. Lee, J. D. Wurtz, “Novel physics arising from phase transitions in biology” *J. Phys. D: Appl. Phys.* **2019**, *52*, 023001.

[5]	A. W. Fritsch, A. F. Diaz-Delgadillo, O. Adame-Arana, C. Hoege, M. Mittasch, M. Kreysing, M. Leaver, A. A. Hyman, F. Jülicher, C. A. Weber, “Local thermodynamics govern formation and dissolution of Caenorhabditis elegans P granule condensates” *Proceedings of the National Academy of Sciences* **2021**, *118*, e2102772118.
